# A Rare but Deadly Complication of Transcatheter Aortic Valve Replacement

**DOI:** 10.7759/cureus.29530

**Published:** 2022-09-24

**Authors:** Saurabhkumar M Limani, Jonathan D Roberts, Nayan K Desai, Sundermurthy Yamini

**Affiliations:** 1 Detartment of Hospital Medicine, Sanford Health, Bismarck, USA; 2 Department of Internal Medicine, University of North Dakota School of Medicine and Health Sciences, Bismarck, USA; 3 Department of Cardiology, Sanford Health, Bismarck, USA

**Keywords:** pci after tavr, tavr, stemi after tavr, delayed coronary obstruction in tavr, tavr complication

## Abstract

We present a rare case of delayed coronary artery obstruction following a transcatheter aortic valve replacement (TAVR). Interestingly, the patient did not meet the criteria for traditionally recognized risk factors for delayed coronary obstruction. This case piques interest as to whether the severity of calcification on aortic valve leaflets plays any role in coronary obstruction post transcatheter aortic valve replacement. There is no consensus as to the optimal approach to investigation and revascularization in patients with delayed coronary obstruction. We report a case with successful emergent revascularization of the left main coronary artery following transcatheter aortic valve replacement.

## Introduction

Transcatheter aortic valve replacement (TAVR) was first described in 2002 as the catheter-based implantation of a crimped valve into the stenotic native aortic valve using an antegrade transapical approach. It was presented as an alternative treatment method to surgical aortic valve replacement (SAVR) for patients who have high or prohibitive surgical risks [1,2]. Since 2002, TAVR use has been rapidly expanding, driven by the growth of the elderly population and a concomitant increase in the prevalence of age-related aortic stenosis (AS). Growing randomized control trials supporting TAVR in intermediate-risk and low-risk populations have led to an exponential increase in the procedural volume of TAVR [3]. While TAVR is less invasive than open heart surgery, it is still associated with complications, which include death, aortic rupture or dissection, pericardial tamponade, acute coronary obstruction, stroke, and flow-limiting peripheral arterial dissection. The risk of delayed coronary artery obstruction following TAVR is low (less than 1%) but carries lethal consequences [4].

As the TAVR procedure becomes more prevalent, there will be an increased incidence of coronary artery obstruction after TAVR. Thus, emergency percutaneous coronary intervention (PCI) will become more common. This brings new technical challenges to interventional cardiologists where the design, material, and anatomical orientation of various implanted valves may impact the feasibility and safety of engaging coronary arteries for intervention. There is currently no consensus as to the optimal approach to investigation and revascularization in these patients [5,6]. Therefore, we hereby report a case of successful emergent revascularization of the left main coronary artery using the snorkel technique.

## Case presentation

This case involves a 76-year-old man with a known history of symptomatic severe bicuspid aortic valve stenosis with dizziness and chronic fatigue. He also had a history of paroxysmal atrial fibrillation and chronic diastolic congestive heart failure.

**Figure 1:**
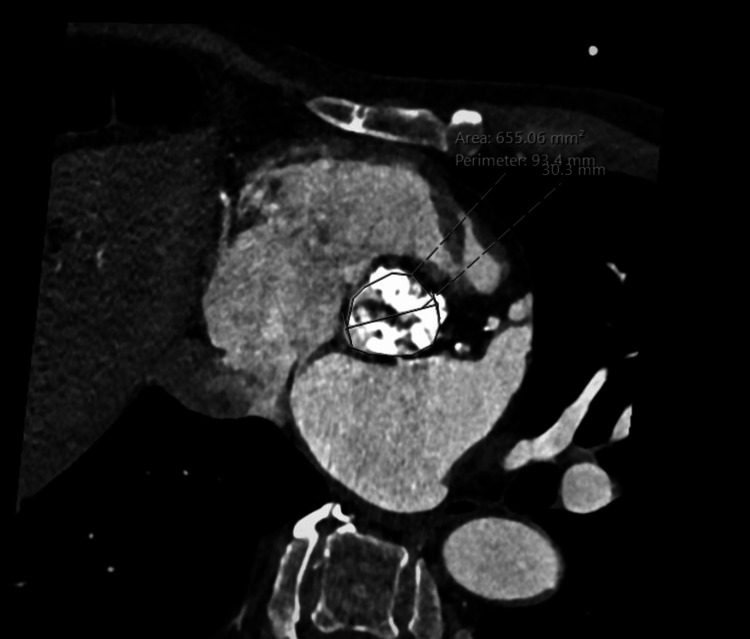
Mean aortic annulus diameter 30.3 mm

**Figure 2:**
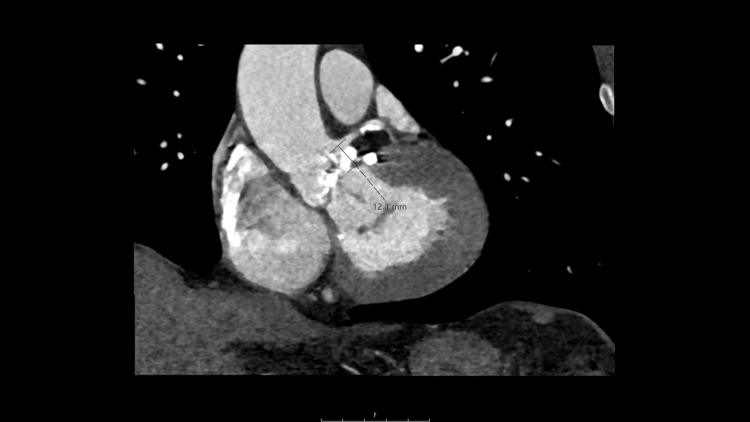
Coronary artery ostial height 12.1 mm

The outpatient pre-procedural evaluation began with a 12-lead electrocardiogram (EKG), which showed a normal sinus rhythm, left ventricular hypertrophy, and no conduction abnormality. A transthoracic echocardiogram demonstrated normal right and left ventricle size, normal left ventricular ejection fraction, and severe aortic valve stenosis. The aortic valve had a peak gradient of 105.3 mmHg and a mean gradient of 63 mmHg. A coronary angiogram showed moderate nonobstructive coronary artery disease within the middle segment of the right coronary artery. This segment had 50% stenosis and an instantaneous wave-free ratio (IFR) of 0.91. An EKG-gated CT angiogram and 3D MIP reconstructions of the chest were performed. Additional 3D images of the aorta were also collected. The aortic valve was noted to be severely calcified with an annular diameter of 30.3 mm, as shown in Figure 1. As seen in Figure 2, the distance from the annulus to the origin of the left main coronary artery was 12.1 mm. The right coronary ostium was at a height of 11 mm above the annulus. Careful analysis of the CT reconstruction revealed adequate coronary Ostia heights and adequate dimensions of the sinuses of Valsalva. He was designated high-risk by multidisciplinary evaluation and was scheduled for an elective TAVR.

Upon admission, he reported a six-month history of dizziness and activity-limiting fatigue. Physical examination revealed a heart rate of 62 beats per minute (bpm), blood pressure of 143/62 mmHg, the absence of the A2 component of the heart sound, and a 4/6 systolic ejection murmur radiating to the carotid arteries. The lungs were clear to auscultation, the abdomen was soft and not tender to palpation, and there was no peripheral edema.

Moderate anesthesia care (MAC) with local anesthesia was performed, and via femoral approach, he underwent a transcatheter aortic valve replacement using an Evolut PRO Medtronic self-expanding 34 mm TAVR valve. The procedure was complicated by proximal embolization of the valve on the left side with a moderate to severe perivalvular leak. There was no coronary artery obstruction on the angiogram, and the EKG did not show any ischemic changes. Using the snare technique, the valve was pulled into the ascending aorta. Later, a second 34 mm Evolut PRO valve was deployed with excellent angiographic results. The valve appeared to be seated at approximately +2. Post-deployment ascending aortography showed traces to mild perivalvular aortic regurgitation. Post-TAVR hemodynamics showed an aortic pressure of 127/65 mmHg, left ventricular end-diastolic pressure (LVEDP) of 10 mmHg, a mean valve gradient of 27 mmHg, and sinus rhythm. Because the mean gradient was more than 20 mmHg, balloon angioplasty was performed with a 26 mm balloon. Hemodynamics post balloon angioplasty showed a mean gradient of 13 mmHg. A post-TAVR transthoracic echocardiogram revealed an appropriate position and function of the valve, no significant perivalvular leak, no pericardial effusion, and normal LV function. The patient was transferred to the post-anesthesia care unit (PACU) and later to the telemetry unit in stable hemodynamic condition.

Six hours postoperatively, the patient started complaining of ten out of ten chest pain with pleuritic radiation. He was diaphoretic, hypotensive, and in sinus bradycardia with a heart rate of 38 bpm. He was given atropine with an improvement in sinus bradycardia and blood pressure. At this point, multiple differential diagnoses were entertained, including aortic dissection, pulmonary embolism, spontaneous pneumothorax, acute coronary syndrome (ACS), and pericardial effusion. A stat-limited echocardiogram was performed, which ruled out pericardial effusion and showed normal TAVR valvular function. A stat CTA of the chest showed no pulmonary embolism or pneumothorax. An EKG showed evidence of acute myocardial infarction with ST elevation in lead aVR and depression in leads V2 and V3. The patient was rushed to the cardiac catheterization lab for an emergent coronary angiogram.

**Figure 3:**
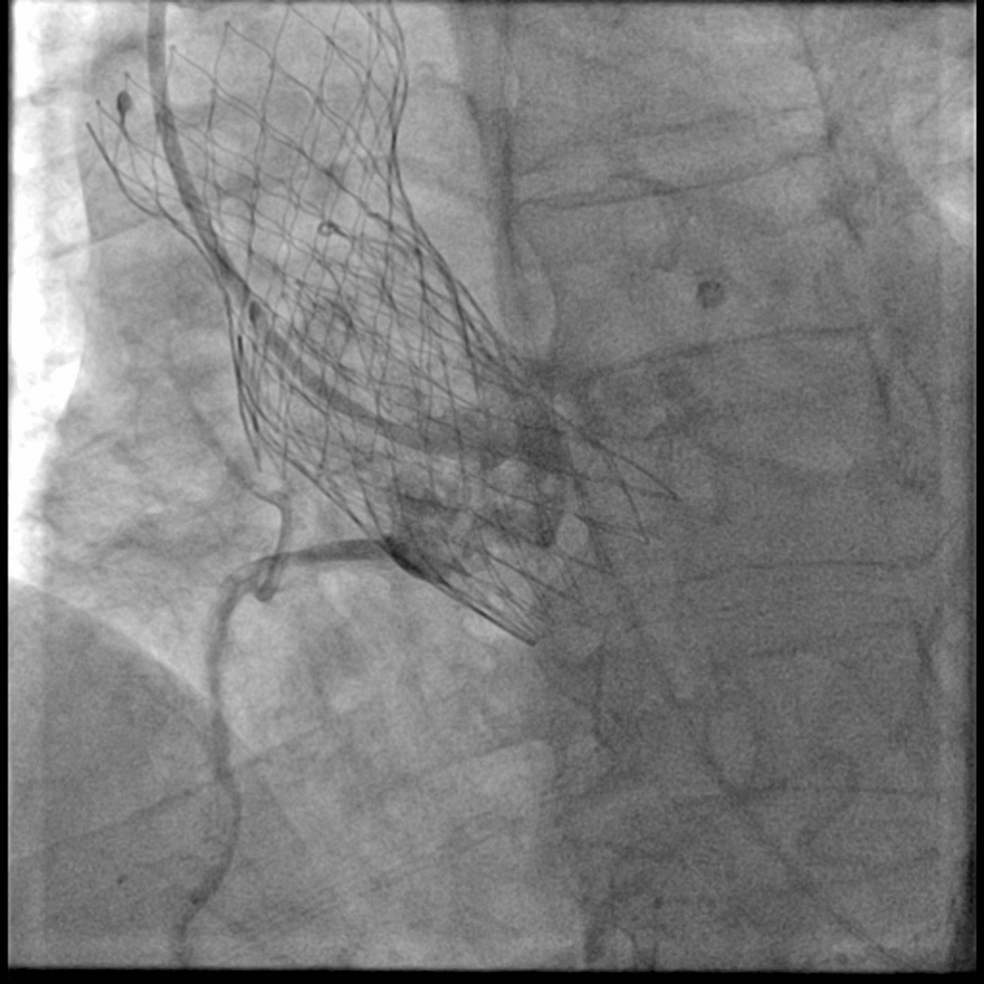
Complete obstruction of left main coronary artery by bulk of calcification from aortic valve leaflet

**Figure 4:**
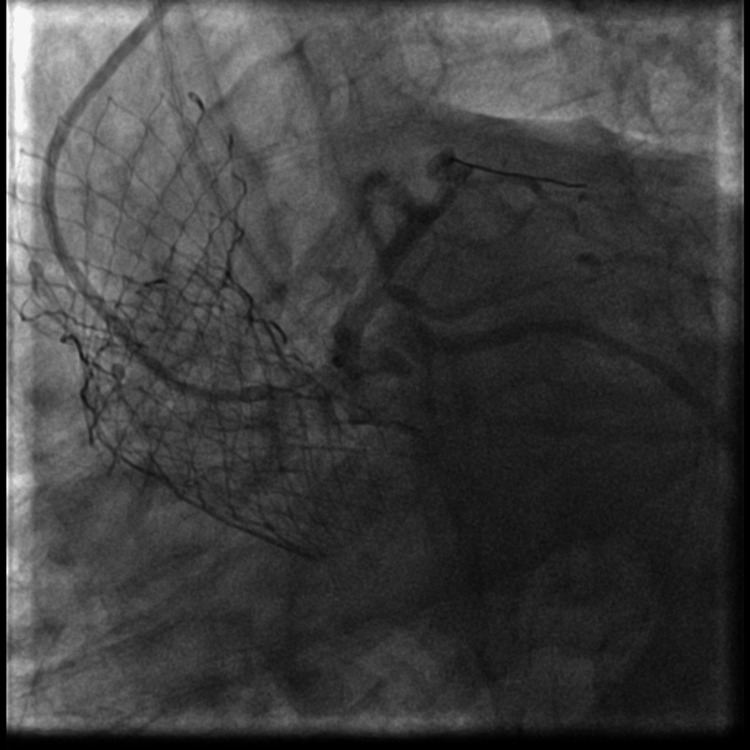
Percutaneous coronary intervention of left main coronary artery using Snorkeled technique

The left main ostia was found to be externally compressed by a heavily calcified native aortic valve leaflet as seen in Figure 3. PCI was performed with an Onyx resolute 5.0 mm × 22 mm drug-eluting stent (DES) that was overlapped with an Xience 4.0 mm × 18 mm DES. These stents were placed using the snorkel technique from the ascending aorta into the middle segment of the left main coronary artery. The stents were dilated with a 6.0 NC balloon with excellent results and thrombolysis in myocardial infarction (TIMI) grade 3 flow as shown in Figure 4. The second stent was placed with the aim of increasing the radial strength of the stent. An EKG showed improvements in ST changes in aVR and V2-V3. After the procedure, the patient reported improvement in chest pain, which was now a three out of ten in severity.

The patient was transferred to the intensive care unit (ICU) for close monitoring overnight. The next day, he began a one-month treatment plan with aspirin, Prasugrel, and Apixaban. Twenty-four hours after the PCI, he reported no chest pain, and no arrhythmias were noted on telemetry; therefore, the patient was transferred out of the ICU to the telemetry floor. At 48 hours post PCI, he continued to deny any chest pain, telemetry did not show any arrhythmias, and ambulating without difficulty; therefore, he was discharged home with plans for outpatient follow-up.

At a follow-up visit on day 11, he was noted to have weight gain, elevated jugular venous pressure, and lower extremity swelling. An EKG showed atrial flutter with a rapid ventricular response. The patient had no chest pain. His metoprolol was increased, and Torsemide was added to his regimen. On day 18, after hospital discharge, he was readmitted with shortness of breath. An EKG showed atrial flutter with a rapid ventricular response and biventricular heart failure. In the ICU, he was started on a milrinone and Lasix drip. On the echocardiogram, the ejection fraction was noted to be 30% (a decrease from 55% previously). The TAVR valve appeared to be in an appropriate position with trace perivalvular regurgitation, and the gradient across the valve was 9 mmHg. Cardiomyopathy was thought to be due to tachycardia-induced given his global hypokinesia. The volume status was optimized, the milrinone drip was weaned off, guideline-directed medical therapy was initiated, and he was discharged home. At two-day, two-week, and six-week post-discharge follow-up visits from the second hospitalization, he was doing well, riding a bicycle, and participating in cardiac rehab.

## Discussion

Delayed coronary obstruction after TAVR is a rare complication that was first described in 2006 [7]. Multiple studies report an incidence of less than 1%, with approximately 90% of cases involving the left coronary artery [4,8]. DCO occurs more commonly after valve-in-valve procedures when self-expandable valves are used and within 24 hours of the procedure [4]. The most common mechanisms for DCO are displacement of a calcific native valve leaflet or surgical valve leaflet. Less common causes include leaflet avulsion or calcific embolization, aortic root dissection, hematoma extending to the coronary ostium, or embolization of a thrombus located at the TAVR valve [9]. DCO is more common in women, with studies reporting 76% to 83% of DCO cases occurring in females [4,10]. This may be due to anatomical differences in the aortic root, sinus of Valsalva (SOV), and coronary ostium. Studies have found that women tend to have smaller aortic roots and lower coronary ostia heights [9,11].

The presentation of DCO varies, with signs and symptoms including angina, hypotension, EKG changes, and cardiac arrest. Studies have reported cardiac arrest or persistent hypotension as the most common presenting symptom [4,8]. Emergent PCI remains the mainstay of treatment in this situation. Percutaneous intervention has a reported success rate of 68% to 91% [4,8,10,12]. Despite the high PCI success rate, DCO is still associated with high mortality rates. Various systematic reviews report 30-day mortality rates of between 8.3% and 50% [4,8,10,12]. There is also a reported increased mortality rate with sooner onset of DCO [4].

The risks that accompany percutaneous coronary intervention are even greater with DCO post-TAVR. Risks include infection, stroke, death, and difficult access through the implanted valve. A recent cohort study of patients with ACS post-TAVR reported coronary artery access issues in 2.5% of coronary angiography procedures [13]. Despite the increased risks, PCI has been shown to decrease the all-cause mortality rate of DCO [13]. Another measure for the effective treatment of DCO is having access to emergent cardiovascular surgery. In one study, conversion to open heart surgery occurred in 8% of PCI attempts [9].

DCO occurs most commonly with self-expandable valves and with valve-in-valve procedures. Other risk factors include female sex, older age, and no previous coronary artery bypass graft [4,8]. Identified anatomical risk factors include a smaller aortic annulus (diameter <22 mm), a smaller sinus of Valsalva (diameter < 28 mm), and a shorter coronary ostia height (<10 mm) [4,8]. These anatomical risk factors increase the probability that, upon deployment, the prosthetic valve will force the native leaflets over the ostium. It is worth noting that a large multicenter registry has found that 20% of left main artery occlusions occurred in patients with a left coronary height greater than 12 mm and 35% of occlusions occurred in patients with a SOV diameter greater than 30 mm [4,10]. An additional predictor of coronary artery obstruction is curved leaflet length in relation to sinus dimension [7]. One study suggests that a ratio >1 between leaflet length and coronary sinus height could be a new and useful predictor of coronary artery occlusion [7]. In our patient, we were not able to measure the left coronary leaflet length due to heavily calcified and fused leaflets.

Interestingly, the patient described did not meet several of the recognized criteria for delayed coronary obstruction. The patient was a younger male who did not have high-risk anatomical dimensions. His SOV measurements were 33 mm × 32 mm × 32 mm, his left main ostium height was 12.1 mm, and his average aortic annulus diameter was 30.3 mm. The patient had none of the identified risk factors for DCO.

In the case described, a calcific nodule on the native valve leaflet was the culprit of DCO. Studies have suggested heavy calcification as a risk factor for DCO [4,11,14]. In the patient presented, a bicuspid aortic valve could have also contributed to DCO. A study of bicuspid aortic valve patients found the incidence of DCO after TAVR to be 0.1%, similar to that found in other studies including both bicuspid and tricuspid valve patients [15]. However, it has been shown that bicuspid aortic valves have a higher calcium burden compared to tricuspid valves [16]. There is a clear disconnect between the expected rate of DCO in bicuspid aortic valve patients and the actual rate. A higher rate of DCO is expected among bicuspid aortic valve patients given their association with a higher calcium burden. This case highlights the increased calcium burden in bicuspid aortic valve patients and the increased risk of delayed coronary obstruction.

## Conclusions

DCO following TAVR is a rare but fatal complication with a high in-hospital mortality rate. It can occur intra-operatively or post-operatively, with the highest incidence occurring within the first 24 hours post-operatively. In the case described, DCO occurred within six hours. The most common presenting symptoms include persistent hypotension, angina, bradycardia, and cardiac arrest. Since PCI is a feasible and successful treatment option in most cases of DCO, the provider should have a low threshold for performing coronary angiography when a coronary obstruction is suspected. When there are no ischemic changes noted on an EKG, other complications such as pericardial effusion with tamponade, spontaneous pneumothorax, and aortic dissection should also be ruled out. In addition to the readily identified risk factors of DCO, native valve leaflet calcification and its severity could also be an independent risk factor. It is imperative that multidisciplinary evaluation not only consider leaflet calcification but also the variability of bicuspid aortic valves and their association with increased calcium burden.
